# Supplementary material for: Large-Area Biocompatible Random Laser for Wearable Applications
Source: Nanomaterials (Basel). 2021 Jul 12;11(7):1809. doi: 10.3390/nano11071809 (PMC8308224; doi:10.3390/nano11071809)
Supplement: Supplementary file 1 [file nanomaterials-11-01809-s001.zip › nanomaterials-1278868-supplementary.pdf]

nanomaterials MDPI

Supporting Information

Large-Area Biocompatible Random Laser for Wearable Applications

Kun Ge [1], Dan Guo [1], Xiaojie Ma [1], Zhiyang Xu [1], Anwer Hayat [1], Songtao Li [2] and Tianrui Zhai [1,*]

1 Faculty of Science, College of Physics and Optoelectronics, Beijing University of Technology, Beijing 100124, China; GEKUN@emails.bjut.edu.cn (K.G.); dguo@bjut.edu.cn (D.G.); xiaojiema@emails.bjut.edu.cn (X.M.); xu.zhiyang@hotmail.com (Z.X.); anwerhayatnoor@gmail.com (A.H.)
2 Department of Mathematics & Physics, North China Electric Power University, Hebei 071000, China; songtaoli2001@126.com
* Email: trzhai@bjut.edu.cn

CONTENTS

1. The PL spectra at different pump positions.
2. The PL spectra without Au NR.
3. The wearable sensor is transferred to the palm.
4. The isopropanol response of polymer film.

1. The PL spectra at different pump positions

A large-area coherent random laser is fabricated by spin-coating and lift-off technique. The area of the polymer film can be up to over 100 cm^2. The PL emission spectra of the coherent random laser are at different pump positions on the large-area polymer film as shown in Figure S1a–b. In the experiment, the ten groups of PL spectra are recorded at different pump position from 1 to 10. We set first pump position as position 1, and the distance between two pump positions is about 1 cm. The intensity of random laser changes little, and the full width at half maximum of coherent random laser have hardly changed, the FWHM is about 0.4 nm.

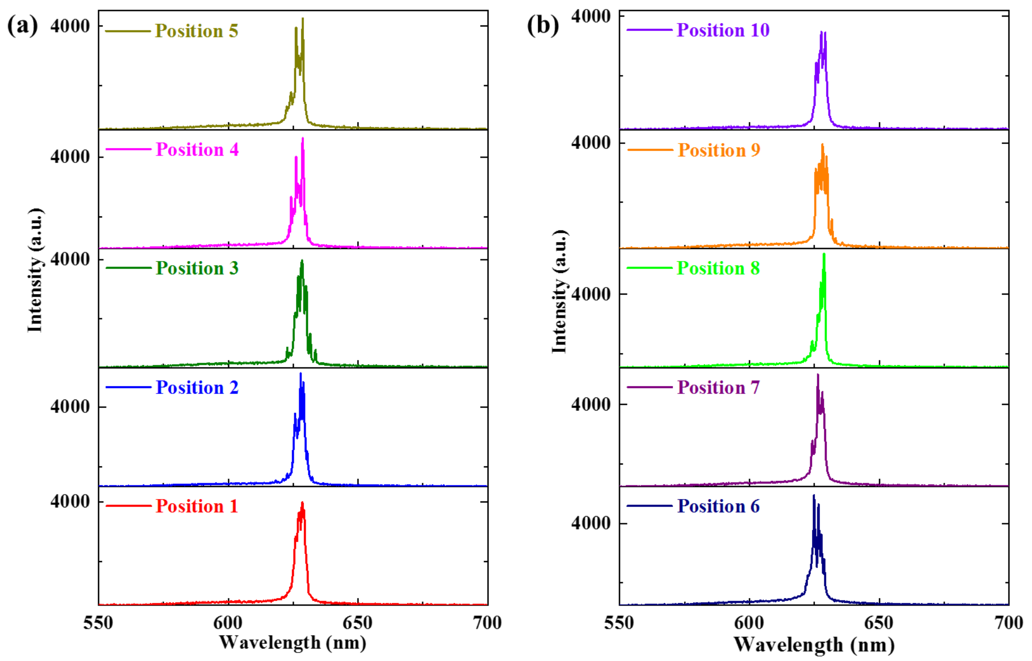

Figure S1. The PL emission spectra of the coherent random laser are at different pump positions on the large-area polymer film. (a) and (b) The different pump position from 1 to 10. The distance between two pump positions is about 1 cm.

2. The PL spectra without Au NRs.

Figure S2 demonstrates the PL spectra without Au nanorods distributing into the polymethyl methacrylate film. The PL emission of random lasing is at pump fluences from 20 μJ/cm² per pulse to 159 μJ/cm² per pulse (Figure S2a). The threshold is about 88 μJ/cm² per pulse, showing curvature change point of blue solid line in Figure S2b.

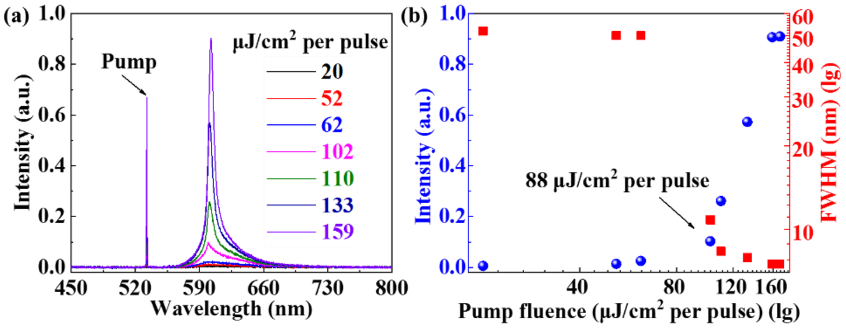

Figure S2. The spectra characterization of the random laser without Au NRs. (a) The emission spectra of random lasing are at different fluences. (b) The output intensity of the random laser is as a function of the pump fluences, indicating the threshold is about 88 μJ/cm2 per pulse. The FWHM of the output lasing is as a function of the pump fluence.

3. The wearable sensor is transferred to the palm

The PL spectrum of random laser for wearable sensor is shown in Figure S3. The polymer film is flexible and transferable, which can be transferred to human skins to serve as a sensor. Pumped by a nanosecond laser with wavelength of 532 nm, Figure S3 provides the optical spectrum with the polymer film transfers to the palm in the experiment. The image shows the polymer film on the palm (inset in Figure S3).

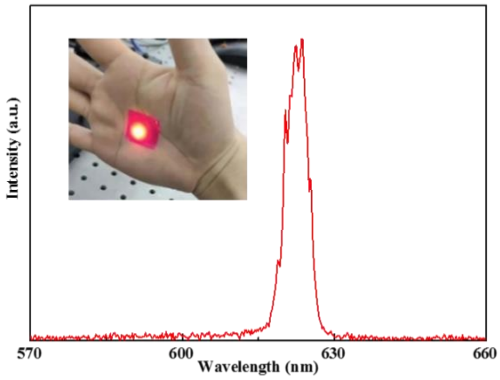

Figure S3. The optical spectrum shows that the polymer film is transferred to the palm when pumped by a nanosecond laser with wavelength of 532 nm. Inset: the image on the palm.

4. The isopropanol response of polymer film.

As a comparative experiment, we explore the wavelength shift of the random laser when the polymer film is exposed various isopropanol vapor. The PL emission spectra is blue-shifted when the gas concentration increases in Figure S4a. The experimental blue-shifted is as a function of refractive index. When the RH of isopropanol increases, the equivalent refractive index decreases for the polymer film will absorb the isopropanol molecules. The blue-shifted of the spectrum of random laser is caused by the decrease of the equivalent refractive index. In addition, the concentration of gain

material will decrease with isopropanol molecules adsorbed on polymer film, which will cause the blue-shifted of the lasing mode. Figure S4b provides plots of shift of emissions peaks is as a function of relative humidity ranging from 0% to 70%.

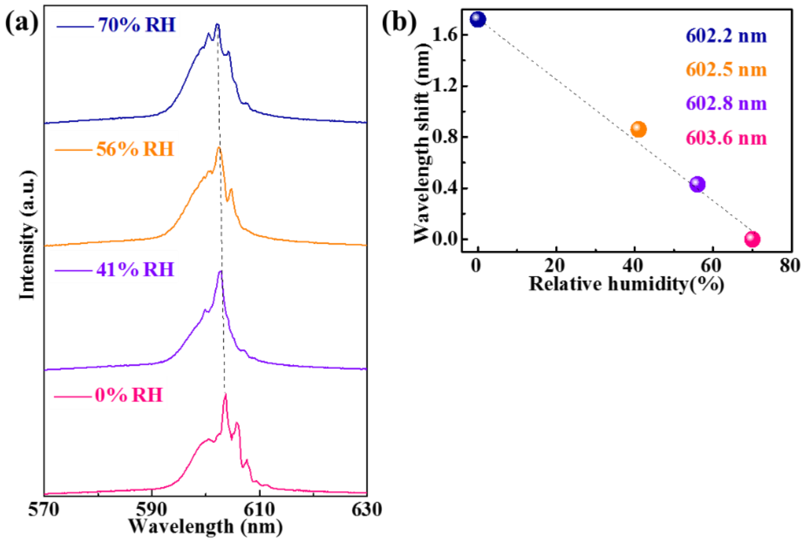

Figure S4. The coherent random laser based on polymer film for isopropanol gas sensing. (a) The PL emission spectra is blue-shifted under exposure various isopropanol vapor. (b) Plots of shift of emissions peaks as a function of relative humidity ranging from 0% RH to 70% RH.
